# Wrist Flexor Spasticity and Hemiplegic–Contralateral Median Nerve Latency Asymmetry After Stroke: A Bilateral Nerve Conduction and Ultrasound Study

**DOI:** 10.3390/diagnostics16071088

**Published:** 2026-04-03

**Authors:** Ki-Hyeok Ku, Seongmin Choi, Kyung Chul Noh, Eo Jin Park

**Affiliations:** 1 Department of Orthopedic Surgery, Kyung Hee University College of Medicine, Kyung Hee University Hospital at Gangdong, Seoul 05278, Republic of Korea; 2 Department of Physical and Rehabilitation Medicine, Kyung Hee University College of Medicine, Kyung Hee University Hospital at Gangdong, Seoul 05278, Republic of Korea; 3 Department of Neurology, Kyung Hee University College of Medicine, Kyung Hee University Hospital at Gangdong, Seoul 05278, Republic of Korea; 4 Department of Rehabilitation Medicine, Kyung Hee University College of Medicine, Kyung Hee University Hospital at Gangdong, Seoul 05278, Republic of Korea

**Keywords:** stroke, hemiplegia, median nerve, spasticity, nerve conduction study, ultrasonography

## Abstract

**Background/Objectives:** The paretic wrist after stroke may exhibit median nerve conduction abnormalities, but factors underlying hemiplegic–contralateral asymmetry remain uncertain. We compared electrodiagnostic and ultrasonographic wrist measures between sides and assessed predictors of side-to-side differences in distal motor latency (ΔDML) and distal sensory latency (ΔDSL). **Methods:** We retrospectively analyzed 85 patients with stroke. Distal motor latency (DML), distal sensory latency (DSL), wrist-to-forearm ratio (WFR), and median nerve inlet cross-sectional area (CSA) were measured bilaterally. Paired *t*-tests evaluated hemiplegic–contralateral differences, and Wilcoxon signed-rank tests were performed as sensitivity analyses. Multivariable linear regression with robust (HC3) standard errors modeled ΔDML as the primary outcome and ΔDSL as the secondary outcome, with wrist flexor spasticity (Modified Ashworth Scale, MAS) specified a priori as the primary explanatory variable; extended models additionally included ΔWFR. Sensitivity analyses re-specified MAS as an ordered category, and complementary linear mixed-effects models using raw bilateral latency values were fitted to assess the robustness of Δ-based modeling. **Results:** The hemiplegic side showed higher DML (5.51 ± 0.79 vs. 4.81 ± 0.42 ms; mean difference 0.694; *p* < 0.001), DSL (4.51 ± 0.88 vs. 3.66 ± 0.45 ms; mean difference 0.852; *p* < 0.001), WFR (1.21 ± 0.30 vs. 1.07 ± 0.16; *p* = 0.008), and CSA (11.16 ± 3.67 vs. 9.69 ± 2.04 mm^2^; *p* = 0.032). MAS was associated with ΔDML (β = 0.336; *p* < 0.001) and ΔDSL (β = 0.238; *p* = 0.015). ΔWFR remained significant for ΔDML (β = 1.314; *p* < 0.001) and ΔDSL (β = 1.371; *p* = 0.001), improving adjusted R^2^ from 0.251 to 0.370 for ΔDML and from 0.142 to 0.253 for ΔDSL. Findings remained directionally consistent when MAS was modeled as an ordered category. Complementary mixed-effects models using raw bilateral latency values showed significant hemiplegic-side-by-MAS interactions for both DML (β = 0.425; 95% CI 0.275 to 0.575; *p* < 0.001) and DSL (β = 0.366; 95% CI 0.195 to 0.537; *p* < 0.001). **Conclusions**: In chronic stroke hemiplegia, median nerve latencies and wrist morphology may differ between sides. Wrist flexor spasticity and side-to-side increases in WFR may be independently associated with greater latency asymmetry. These interlimb latency differences should be interpreted as physiological markers of side-to-side median nerve involvement at the wrist rather than as stand-alone diagnostic criteria for carpal tunnel syndrome.

## 1. Introduction

Wrist flexor spasticity is commonly observed during routine neurological and rehabilitation evaluations, and chronic stroke hemiplegia is typically accompanied by aberrant muscle tone and persistent motor dysfunction [1,2,3]. In clinical practice, central nervous system injury is usually invoked to explain functional limitations of the paretic upper limb; however, peripheral nerve status at the wrist may also contribute to hand dysfunction, pain, and sensory symptoms [2,4,5]. Because anatomical and electrophysiological techniques may help characterize limb neurological state beyond bedside motor examination, objective evaluation of the median nerve at the wrist may be clinically important [6,7].

Interpretation of wrist-level median nerve abnormalities should also consider relevant clinical context and patient-level risk factors, including metabolic comorbidities [8]. In this context, standardized quantitative measures of median nerve function are provided by nerve conduction studies (NCS). Specifically, when assessing median nerve function at the wrist, latency-based measures such as distal motor latency (DML) and distal sensory latency (DSL) are frequently employed [6,9]. By providing wrist-level morphological indices, such as the median nerve cross-sectional area at the carpal tunnel inlet (CSA inlet) and the wrist-to-forearm ratio (WFR), which can act as a within-person reference for potential nerve enlargement at the wrist relative to the forearm, neuromuscular ultrasound may supplement NCS [6,7,10]. Side-to-side differences may arise following unilateral motor impairment, as limb posture and spasticity have been proposed as potential contributors to peripheral nerve alterations at the wrist in post-stroke populations [11,12,13].

However, several uncertainties remain. First, the extent to which wrist flexor spasticity correlates with hemiplegic–contralateral asymmetry in median nerve conduction latency has not been fully established, particularly when using within-subject comparisons in which each patient serves as their own control [5,11,14]. Second, although ultrasound can identify variations in wrist morphology, it is unclear whether side-to-side differences in ultrasound-derived indices—particularly WFR—are associated with latency asymmetry or adequately explain electrophysiological side-to-side differences in chronic stroke hemiplegia [15,16,17]. Given that spasticity may be evaluated at the bedside and that ultrasound is becoming more widely available as an NCS adjunct, addressing these concerns is clinically relevant [6,18].

A paired bilateral design offers a practical approach to investigating these issues. In addition to highlighting limb-specific differences consistent with unilateral impairment, within-subject hemiplegic–contralateral comparisons can help minimize confounding by stable patient-level characteristics (e.g., age, metabolic comorbidities, or generalized sensitivity to neuropathy) [19,20]. Furthermore, combining NCS and ultrasonography within the same paired framework may provide complementary anatomical and functional perspectives on the condition of the median nerve at the paretic wrist [6,21].

Accordingly, this study aimed to (1) characterize hemiplegic–contralateral asymmetry in wrist ultrasound measures (WFR and CSA inlet) and median nerve NCS latencies (DML and DSL) in chronic stroke hemiplegia; and (2) evaluate whether greater latency asymmetry is associated with greater wrist flexor spasticity. The primary outcome was ΔDML, the secondary outcome was ΔDSL, and analyses involving ultrasound-derived asymmetry variables (ΔWFR and ΔCSA) were prespecified as secondary/exploratory analyses intended to determine whether local wrist morphology provides additional explanatory information beyond spasticity alone. Throughout the study, interlimb latency asymmetry was interpreted as a physiological marker of side-to-side wrist-level median nerve involvement rather than as a stand-alone diagnostic criterion for carpal tunnel syndrome.

## 2. Materials and Methods

### 2.1. Participants

Between January 2019 and February 2025, we retrospectively reviewed adult patients with chronic post-stroke hemiplegia who presented to the Department of Rehabilitation Medicine at Kyung Hee University Hospital, Gangdong. Patients were eligible if they met all of the following criteria: (1) age ≥19 years; (2) diagnosis of ischemic or hemorrhagic stroke with unilateral hemiplegia; (3) time since stroke onset ≥3 months; (4) availability of bilateral median nerve NCS data at the wrist, including DML and DSL; (5) availability of bilateral wrist ultrasonography data, including WFR and median nerve CSA inlet. Wrist flexor spasticity was assessed using the Modified Ashworth Scale (MAS). NCS and ultrasonography were completed within 30 days of each other.

Exclusion criteria included conditions that could significantly compromise the interpretation of median NCS (e.g., cervical radiculopathy or brachial plexopathy affecting the tested limb), severe polyneuropathy that precluded reliable differentiation of focal median neuropathy at the wrist, prior use of a wrist splint or brace on the affected upper limb, and a history of carpal tunnel surgery or previous invasive treatment for carpal tunnel syndrome [22,23].

### 2.2. Clinical Variables

Collected demographic and clinical variables included age, sex, body mass index (BMI), diabetes mellitus status, and time since the stroke onset (months). Stroke severity and functional status were assessed using routinely recorded clinical data from the National Institutes of Health Stroke Scale (NIHSS) and the Korean version of the Modified Barthel Index (K-MBI), respectively [24,25]. Standardized provocative clinical tests for carpal tunnel syndrome, including Phalen’s and Tinel’s signs, were performed as part of routine clinical evaluation; however, these findings were not included in the present analysis because they were not incorporated as predefined study variables in the retrospective dataset.

### 2.3. Spasticity Assessment

During the same evaluation session as ultrasonography and electrodiagnostic testing, a board-certified physiatrist evaluated wrist flexor spasticity using the MAS [26,27]. The examiner passively moved the wrist through its range of motion at a consistent speed and graded resistance to passive stretch according to MAS criteria while the patient was in the supine position and the forearm was supported in a neutral posture [28,29]. MAS scores were handled as ordinal values descriptively. For the primary regression analyses, MAS 1+ was coded as 1.5 as a pragmatic approximation to preserve the ordered progression of the scale and to facilitate coefficient interpretation across adjacent severity levels [30,31,32]. Because MAS is not an interval-scaled measure, sensitivity analyses additionally re-specified MAS as an ordered category.

### 2.4. Nerve Conduction Studies

Bilateral median nerve NCS were performed using a commercial electromyography system (Nicolet EDX, Natus Medical Incorporated, Middleton, WI, USA), according to the institutional standard protocol.

Motor study (median nerve): Motor conduction was assessed by stimulating the median nerve at the wrist and recording with surface electrodes placed over the abductor pollicis brevis [33]. DML (ms) was defined as the onset latency of the motor response following wrist stimulation [33]. The stimulation-to-recording distance was fixed at 8 cm along the nerve course, and the stimulation site was located at the wrist between the flexor carpi radialis and palmaris longus tendons.

Sensory study (median nerve): Sensory conduction was evaluated using an antidromic approach, with surface-adhesive electrodes recording from digit III and stimulation applied to the wrist [34,35]. The distance between the recording electrodes and the stimulation site, measured along the nerve course, was fixed at 14 cm [34,35]. DSL (ms) was defined as the peak latency of the sensory response [34,35]. Digit III was selected because the antidromic median sensory recording at a fixed 14 cm distance is a conventional technique that provides stable and reproducible sensory responses in routine electrodiagnostic practice [9,36].

Because the primary objective of this study was bilateral within-nerve comparison rather than median–ulnar comparative testing within the same hand, a single standardized median sensory protocol was applied to both sides throughout the study period.

Analyses were limited to latency measures (DML and DSL) because the study focused on hemiplegic contralateral variations in conduction delay [37]. Response amplitudes were excluded because they are more susceptible to technical factors, such as electrode placement, and to changes related to paretic muscle status in routine clinical recordings [38,39]. DML and DSL were measured bilaterally for both the hemiplegic and contralateral limbs.

### 2.5. Ultrasonography

Bilateral ultrasonographic examinations were performed using a high-frequency linear transducer (Philips HD11; Koninklijke Philips Electronics N.V., Amsterdam, The Netherlands, 12–18 MHz). Participants were in a standard position, with the wrist neutral and the forearm supinated. The median nerve was identified in the transverse plane.

CSA inlet: CSA inlet (mm^2^) was measured at the level of the pisiform (carpal tunnel inlet) [40,41]. The CSA was determined by tracing the inner border of the hyperechoic epineurium [40,41].

Forearm CSA and WFR: A second CSA measurement was obtained at the distal forearm, located at a predetermined distance of 12 cm proximal to the distal wrist crease [35,42]. The WFR was calculated as follows:

WFR = CSA inlet/CSA at distal forearm.

At least two CSA measurements were obtained at each site, and the mean value was used for analysis [43].

Derived side-to-side difference variables: Side-to-side differences were calculated as the value on the hemiplegic side minus that on the contralateral side to quantify hemiplegic–contralateral asymmetry:

ΔDML = DML (hemiplegic) − DML (contralateral),

ΔDSL = DSL (hemiplegic) − DSL (contralateral),

ΔWFR = WFR (hemiplegic) − WFR (contralateral),

ΔCSA = CSA inlet (hemiplegic) − CSA inlet (contralateral).

The primary outcome was ΔDML, and the secondary outcome was ΔDSL. Ultrasound-based asymmetry variables (ΔWFR and ΔCSA) were prespecified for secondary/exploratory analyses assessing whether local wrist morphology provided additional explanatory information beyond spasticity. These asymmetry variables were analyzed as physiological markers of side-to-side wrist-level differences and not as stand-alone diagnostic criteria for carpal tunnel syndrome.

### 2.6. Statistical Analysis

Categorical variables are presented as counts and percentages (*n* [%]) and continuous variables as mean ± standard deviation or median [interquartile range], as appropriate. The primary outcome was ΔDML, and the secondary outcome was ΔDSL. Paired comparisons between hemiplegic and contralateral sides were performed for DML, DSL, WFR, and CSA inlet. Cohen’s dz (mean paired difference divided by the standard deviation of the paired difference) was calculated to estimate standardized paired effect sizes. Normality of paired differences was assessed using the Shapiro–Wilk test. Paired *t*-tests were used as the primary method for paired comparisons, and Wilcoxon signed-rank tests were performed as sensitivity analyses.

For regression analyses, multivariable linear regression models were constructed using ΔDML and ΔDSL as dependent variables. Wrist flexor spasticity (MAS) was specified a priori as the primary explanatory variable, while age, sex, BMI, diabetes mellitus status, and duration since onset (months) were included as covariates. A base model including MAS and covariates was first fitted for each outcome, followed by an expanded model that additionally included ΔWFR. In the primary analysis, MAS 1+ was coded as 1.5 as a pragmatic approximation to preserve the ordered progression of the scale. Because MAS is ordinal rather than interval-scaled, sensitivity analyses re-specified MAS as an ordered category corresponding to MAS 0, 1, 1+, 2, 3, and 4.

To assess the robustness of Δ-based modeling, complementary linear mixed-effects models using raw bilateral latency values were fitted separately for DML and DSL, with side (hemiplegic vs. contralateral), MAS, and a side-by-MAS interaction specified as fixed effects, a subject-level random intercept, and the same covariates as in the primary models. Collinearity in the expanded Δ models was assessed using correlation coefficients and variance inflation factors (VIFs). Because the dataset was retrospective and cross-sectional, mediation was not formally modeled; instead, exploratory interaction terms between MAS and ΔWFR were examined to assess possible effect modification. Residual distributions and heteroscedasticity were assessed using standard diagnostic procedures, and robust (HC3) standard errors were applied for inference in the linear regression models. Model performance was summarized using adjusted R^2^. Statistical significance was defined as *p* < 0.05 (two-sided). The primary analyses were performed using SPSS version 20.0 for Windows (IBM Corp., Armonk, NY, USA), and supplementary sensitivity, collinearity, and mixed-effects analyses were performed using R (version 4.2.3; R Foundation for Statistical Computing, Vienna, Austria).

### 2.7. Ethics Approval

This study was approved by the Institutional Review Board of Kyung Hee University Hospital at Gangdong (approval number: 2026-02-025). The requirement for informed consent was waived due to the retrospective nature of the study and the use of de-identified data.

## 3. Results

### 3.1. Participant Characteristics

A total of 85 patients with chronic post-stroke hemiplegia were included (Table 1). The mean age was 60.16 ± 11.36 years; 48 participants (56.5%) were male, and 37 (43.5%) were female. Mean BMI was 25.27 ± 3.28 kg/m^2^, and 20 patients (23.5%) had diabetes mellitus. The median duration since stroke onset was 13 months [6–28]. The median NIHSS was 6 [4–9], and the median K-MBI was 69 [60–82]. Stroke etiology was ischemic in 61 patients (71.8%) and hemorrhagic in 24 (28.2%). The affected side was left in 53 patients (62.4%) and right in 32 (37.6%). Wrist flexor spasticity, assessed using MAS, was distributed as follows: MAS 0 (30.6%), 1 (21.2%), 1+ (15.3%), 2 (18.8%), 3 (8.2%), and 4 (5.9%).

### 3.2. Hemiplegic–Contralateral Comparisons

In paired comparisons, the hemiplegic side demonstrated longer median nerve latencies than the contralateral side. Both DML and DSL were higher on the hemiplegic side. Ultrasound measures also differed between sides, with greater WFR and larger CSA inlet on the hemiplegic side. Although direct comparison with published normative datasets should be interpreted cautiously because measurement protocols vary across studies, the hemiplegic-side mean latencies and CSA values appeared relatively elevated, whereas contralateral values were closer to expected reference ranges [9,16]. Normality was not rejected for ΔDSL, ΔWFR, or ΔCSA, whereas ΔDML showed modest deviation from normality on the Shapiro–Wilk test (*p* = 0.021). Nevertheless, Wilcoxon signed-rank sensitivity analyses were directionally consistent and remained significant for all paired comparisons (DML, *p* < 0.001; DSL, *p* < 0.001; WFR, *p* < 0.001; CSA inlet, *p* = 0.001) (Table 2).

### 3.3. Associations Between Spasticity and Latency Asymmetry

Regression analyses were performed using side-to-side differences (Δ = hemiplegic − contralateral) as dependent variables (Table 3). In the base model for ΔDML, wrist flexor MAS scores were positively associated with ΔDML. After adding ΔWFR to the model, MAS remained significantly associated with ΔDML, and ΔWFR also showed a positive association. In the extended model, male sex was an additional significant predictor, whereas age, BMI, diabetes mellitus, and duration since onset were not significant.

For ΔDSL, MAS was positively associated with ΔDSL in the base model. After adding ΔWFR, both MAS and ΔWFR remained positively associated with ΔDSL. Age, BMI, diabetes mellitus, duration since onset, and sex were not significant predictors in these models (Table 4).

### 3.4. Model Performance

Model fit improved after including ΔWFR for both outcomes. For ΔDML, the adjusted R^2^ increased from 0.251 to 0.370. For ΔDSL, the adjusted R^2^ increased from 0.142 to 0.253 (Table 5).

### 3.5. Sensitivity and Robustness Analyses

When MAS was alternatively modeled as an ordered category corresponding to MAS 0, 1, 1+, 2, 3, and 4, the association with ΔDML remained significant in both the base model (β = 0.317, 95% CI 0.209 to 0.425; *p* < 0.001) and the expanded model including ΔWFR (β = 0.230, 95% CI 0.110 to 0.349; *p* < 0.001). For ΔDSL, the ordered-MAS sensitivity analysis likewise remained significant in the base model (β = 0.252, 95% CI 0.116 to 0.388; *p* < 0.001) and the expanded model (β = 0.166, 95% CI 0.021 to 0.312; *p* = 0.025). Complementary linear mixed-effects models using raw bilateral latency values showed significant hemiplegic-side-by-MAS interactions for both DML (β = 0.425, 95% CI 0.275 to 0.575; *p* < 0.001) and DSL (β = 0.366, 95% CI 0.195 to 0.537; *p* < 0.001), supporting the robustness of the Δ-based approach. Detailed results of the ordered-category MAS sensitivity analyses and complementary mixed-effects models are provided in Supplementary Table S1.

### 3.6. Collinearity and Exploratory Interaction Analyses

MAS and ΔWFR were modestly correlated (Pearson r = 0.371, *p* < 0.001; Spearman ρ = 0.333, *p* = 0.002), but collinearity in the expanded models was low (VIF for MAS, 1.19; VIF for ΔWFR, 1.20). In exploratory interaction analyses, the MAS-by-ΔWFR term was not significant for ΔDML (β = −0.326, 95% CI −0.848 to 0.197; *p* = 0.222). For ΔDSL, the interaction term was nominally significant (β = −0.605, 95% CI −1.198 to −0.012; *p* = 0.046); because this analysis was exploratory and the study was not powered for interaction testing, this finding was interpreted cautiously and not taken as evidence of a confirmed modifying effect. Detailed collinearity diagnostics and exploratory interaction results are provided in Supplementary Table S2.

## 4. Discussion

Both median nerve electrophysiological tests and ultrasound-based wrist morphological indices demonstrated hemiplegic–contralateral disparities in this retrospective cohort of individuals with persistent post-stroke hemiplegia. The hemiplegic side tended to exhibit longer median nerve latencies when using a paired framework (Δ, defined as hemiplegic minus contralateral), and ultrasound measurements also indicated side-to-side variations at the wrist. Collectively, these findings support the concept that limb-specific characteristics may be associated with detectable asymmetry in median nerve conduction and local wrist morphology in chronic stroke hemiplegia.

In hemiplegic populations, within-patient comparison is a clinically intuitive method [44,45]. Both upper limbs share several systemic variables that influence peripheral nerve conduction, including aging, metabolic comorbidities, and generalized neuropathic susceptibility [46,47]. The Δ-based approach may reduce confounding from stable patient-level factors and better emphasize limb-level differences relevant to unilateral impairment by directly comparing the hemiplegic and contralateral sides within the same individual [48]. However, these findings should be interpreted within the context of routine clinical testing rather than as definitive evidence of a single underlying mechanism, as side-specific technical or positional factors could still contribute to the observed asymmetry [21].

A key study finding was that, in multivariable models, wrist flexor spasticity—measured by MAS—was associated with higher latency asymmetry (ΔDML and ΔDSL). Increased wrist flexor tone frequently corresponds to increased resistance to passive extension and a tendency toward a flexed wrist posture, which may plausibly alter the local environment of the median nerve within the carpal tunnel [49,50]. This association has clinical relevance because MAS is easily accessible during routine rehabilitation assessment. In addition, the paretic wrist may remain in sustained non-neutral positions during therapy and daily activities, potentially affecting local soft-tissue mechanics around the carpal tunnel [49,51,52]. The relationship between MAS and both motor and sensory latency asymmetry is consistent with the hypothesis that greater side-to-side electrophysiological abnormalities at the wrist may accompany more pronounced clinically visible spasticity, although the current design cannot determine directionality [53].

Notably, both ΔDML and ΔDSL were associated with spasticity. Although motor involvement may emerge later or with more severe damage, sensory fibers are often considered more vulnerable to early compression [54,55]. Thus, concurrent motor and sensory latent asymmetry is plausible in a chronic-stage cohort, particularly if positional or limb-level biomechanical factors persist over time [55,56]. Spasticity may therefore function not as a direct cause of changes in the median nerve but as a marker of overall limb severity or altered limb use [57,58]. While residual limb-level confounding cannot be excluded, the association remained robust after adjustment for key clinical covariates.

Ultrasound-based indices provided structural context for the electrophysiological findings. Specifically, WFR was employed as the principal ultrasound indicator to investigate the potential relationship between side-to-side morphological variations and latency asymmetry [21,42]. Both ΔDML and ΔDSL were independently correlated with ΔWFR in extended regression models, and adding ΔWFR enhanced model fit. However, prior studies have not shown a uniform relationship between WFR and electrophysiological severity; therefore, the present ΔWFR finding should be interpreted as complementary morphological information rather than as a deterministic surrogate of NCS severity. This pattern supports the hypothesis that, in addition to clinical spasticity and demographic variables, wrist morphology, as measured using a ratio-based ultrasound measure, may provide additional information pertinent to hemiplegic–contralateral latency asymmetry.

Using WFR in a paired framework offers several advantages. Nerve size at a single level is reflected by the absolute CSA at the inlet, which might differ from person to person, depending on body habitus and other variables [59]. WFR may better highlight localized wrist-level enlargement relative to an internal baseline using a distal forearm reference [40,42]. In stroke cohorts, when inter-individual variability is significant and a within-person “reference” may facilitate interpretation, this could be particularly helpful [42,60]. Crucially, the association between MAS and latency asymmetry persisted after inclusion of ΔWFR, indicating that spasticity and WFR capture overlapping but distinct aspects of limb status. Two possible interpretations are proposed. WFR may reflect structural features not fully captured by bedside tone grading, whereas spasticity likely represents dynamic clinical factors (such as posture, passive resistance, and movement pattern) not fully reflected by static morphology. Overall, the findings support a multifactorial explanation for latency asymmetry rather than a single explanatory pathway [10,42].

The presence of structural asymmetry at the wrist is supported by the observed variation in CSA at the carpal tunnel inlet in paired comparisons. However, CSA and WFR do not always provide redundant information. WFR incorporates a reference site and may more accurately reflect localized swelling relative to baseline nerve size, whereas CSA is an absolute measure at a single level [42,61]. In this study, WFR was emphasized as the primary ultrasound marker for exploratory “morphology contribution” studies, whereas CSA inlet serves as a complementary descriptor of side-to-side structural variations [42]. Accordingly, CSA inlet was retained as a descriptive structural variable rather than entered as a primary predictor in the main regression models.

These findings may be clinically relevant for practitioners managing chronic post-stroke hemiplegia [62]. The observed mean side-to-side differences (ΔDML 0.694 ms and ΔDSL 0.852 ms) are numerically notable; however, they should not be equated directly with conventional carpal tunnel syndrome diagnostic thresholds, which are usually based on absolute distal latencies, symptom profiles, and within-hand comparative techniques rather than hemiplegic–contralateral differences alone [6,10]. Although wrist-level peripheral nerve involvement may coexist and contribute to symptoms or functional impairment, complaints in the paretic limb are often interpreted primarily in relation to central neurological injury [63,64]. Ultrasound-defined alterations in wrist morphology provide additional context, and the observed associations suggest that patients with higher wrist flexor spasticity may be more likely to exhibit greater median nerve latency asymmetry [11,13]. Consequently, a paired evaluation approach combining bilateral NCS and ultrasonography may be helpful when peripheral wrist-level involvement is clinically suspected in the paretic limb, particularly when central impairments alone do not fully account for the presentation [6,21]. Accordingly, the present findings are more appropriately interpreted as physiological markers of side-to-side wrist-level median nerve involvement associated with spasticity and local morphology, rather than as stand-alone diagnostic evidence of carpal tunnel syndrome in every patient [65]. This interpretation is broadly consistent with previous reports suggesting that median nerve involvement may coexist after stroke, while also differing from studies designed primarily for formal carpal tunnel syndrome case definition, which typically rely on within-hand comparative techniques and symptom-based diagnostic frameworks [10,63,64].

Caution is warranted in translating these findings to clinical practice. The current study does not establish a causal relationship between wrist morphology, spasticity, and median nerve conduction asymmetry, nor does it demonstrate that interlimb latency asymmetry alone is sufficient to diagnose carpal tunnel syndrome in all patients [13,66]. Instead, these findings may inform future research aimed at elucidating underlying mechanisms, clarifying clinical impact, and developing systematic evaluation methods.

Several limitations should be considered when interpreting these results. First, this was a retrospective single-center study, which limits causal inference and generalizability. Second, residual limb-level confounding and unmeasured clinical factors may have influenced both ultrasonographic and NCS measures, although the paired hemiplegic–contralateral (Δ-based) approach may mitigate confounding from stable patient-level factors. Hand dominance, symptom severity, detailed sensory complaints, past rehabilitation exposure including prior splinting or orthotic use, therapy intensity, tone-management interventions, and provocative clinical test findings were not included as analyzable covariates in the present retrospective dataset, although these factors may influence limb use, local soft-tissue conditions, symptom interpretation, and generalizability. Third, the findings are best interpreted as predictors of latency asymmetry in this chronic post-stroke population, as the study focused on latency-based NCS outcomes and ultrasound indices rather than a comprehensive electrodiagnostic classification of median neuropathy severity. Accordingly, the present data do not allow us to distinguish demyelination, axonal loss, or technical variability with certainty. Fourth, raw NCS waveform files were not uniformly archived in a publication-ready format across all patients, which precluded inclusion of representative trace figures in the current report. Fifth, non-parametric sensitivity analyses were used to support paired comparisons. Instead of depending on a single inferential technique, interpretation should highlight overall patterns and consistency of direction. Finally, male sex reached statistical significance only in the extended ΔDML model after inclusion of ΔWFR; because this finding was model-specific and was not consistently observed across outcomes, it should be interpreted cautiously rather than as a stable sex effect.

Despite these limitations, this study has several strengths. The within-person paired design partially accounts for systemic factors affecting nerve conduction and provides a clinically intuitive framework for evaluating hemiplegic–contralateral asymmetry. Furthermore, combining ultrasound indices (WFR and CSA) with electrophysiological measures (DML and DSL) offers complementary functional and structural perspectives on the median nerve at the wrist. The use of side-to-side difference variables (Δ) facilitates the assessment of limb-specific relationships in multivariable models and aligns with clinical interpretation in unilateral situations.

Future prospective multicenter studies are required to elucidate the temporal correlations among post-stroke median nerve conduction alterations, ultrasound-defined morphology, and wrist flexor spasticity. Longitudinal designs could determine whether changes in wrist morphology correspond with electrophysiological measurements over time and whether the degree of spasticity predicts the eventual development of latency asymmetry. Interventional studies may evaluate the potential effects of activity-based hand use programs, posture education, or organized tone management techniques on NCS outcomes and ultrasound indices (e.g., WFR). Incorporating standardized symptom assessments, hand dominance, detailed therapeutic exposure, functional hand measures, and longitudinal follow-up would strengthen clinical interpretation, improve generalizability, and identify subgroups most likely to benefit from targeted evaluation.

## 5. Conclusions

In chronic post-stroke hemiplegia, hemiplegic–contralateral differences in median nerve latencies and wrist ultrasound measures were observed. In our models, wrist flexor spasticity was associated with increased latency asymmetry. While adding ΔWFR improved model fit, wrist flexor spasticity and side-to-side variations in WFR were independently associated with motor and sensory latency asymmetry. These findings should be interpreted as physiological markers of side-to-side wrist-level median nerve involvement rather than as stand-alone diagnostic criteria for carpal tunnel syndrome. Prospective studies are needed to clarify the underlying mechanisms, temporal sequence, and clinical implications of this asymmetry.

## Figures and Tables

**Table 1:** Baseline characteristics of the study population.

Characteristic	Value
*N*	85
Age (years)	60.16 ± 11.36
Sex, male	48 (56.5%)
Sex, female	37 (43.5%)
BMI (kg/m^2^)	25.27 ± 3.28
Diabetes mellitus	20 (23.5%)
Duration since onset (months)	13 [6–28]
NIHSS	6 [4–9]
K-MBI	69 [60–82]
Etiology: ischemic stroke	61 (71.8%)
Etiology: hemorrhagic stroke	24 (28.2%)
Affected side: Left	53 (62.4%)
Affected side: Right	32 (37.6%)
MAS wrist flexors, *n* (%)	
0	26 (30.6%)
1	18 (21.2%)
1+	13 (15.3%)
2	16 (18.8%)
3	7 (8.2%)
4	5 (5.9%)

Abbreviations: BMI, body mass index; K-MBI, Korean version of the Modified Barthel Index; MAS, Modified Ashworth Scale; NIHSS, National Institutes of Health Stroke Scale. Values are presented as mean ± standard deviation, median [interquartile range], or *n* (%), as appropriate. MAS 1+ was coded as 1.5 for regression analyses.

**Table 2:** Paired comparisons between hemiplegic and contralateral sides.

Measure	Hemiplegic	Contralateral	Mean Difference (95% CI)	Cohen’s dz	*p*-Value
DML (ms)	5.51 ± 0.79	4.81 ± 0.42	0.694 (0.485–0.903)	0.716	<0.001
DSL (ms)	4.51 ± 0.88	3.66 ± 0.45	0.852 (0.627–1.077)	0.818	<0.001
WFR	1.21 ± 0.30	1.07 ± 0.16	0.140 (0.080–0.200)	0.504	0.008
CSA inlet (mm^2^)	11.16 ± 3.67	9.69 ± 2.04	1.466 (0.708–2.224)	0.417	0.032

Abbreviations: CSA, cross-sectional area; DML, distal motor latency; DSL, distal sensory latency; WFR, wrist-to-forearm ratio; CI, confidence interval.Mean difference denotes hemiplegic side minus contralateral side. Cohen’s dz denotes the standardized mean paired difference divided by the standard deviation of the paired difference. Normality of paired differences was assessed using the Shapiro–Wilk test. Paired *t*-tests are presented; Wilcoxon signed-rank tests were additionally performed as a sensitivity analysis.

**Table 3:** Multivariable linear regression for ΔDML (hemiplegic minus contralateral).

Predictor	Model 1 β (95% CI)	Model 1 *p*	Model 2 β (95% CI)	Model 2 *p*
Age (years)	0.005 (−0.012 to 0.023)	0.569	0.003 (−0.014 to 0.019)	0.727
BMI (kg/m^2^)	0.018 (−0.032 to 0.069)	0.476	0.026 (−0.021 to 0.073)	0.28
Diabetes mellitus	−0.147 (−0.600 to 0.306)	0.525	−0.074 (−0.517 to 0.369)	0.743
Duration since onset (months)	0.006 (−0.003 to 0.016)	0.205	0.007 (−0.002 to 0.017)	0.144
MAS (wrist flexors)	0.448 (0.308 to 0.588)	<0.001	0.336 (0.175 to 0.496)	<0.001
Male sex	0.323 (−0.029 to 0.675)	0.072	0.408 (0.071 to 0.746)	0.018
ΔWFR (hemi–contra)			1.314 (0.652 to 1.977)	<0.001

Abbreviations: BMI, body mass index; CI, confidence interval; DML, distal motor latency; MAS, Modified Ashworth Scale; WFR, wrist-to-forearm ratio. Δ indicates hemiplegic side minus contralateral side. Robust (HC3) standard errors were used for inference.

**Table 4:** Multivariable linear regression for ΔDSL (hemiplegic minus contralateral).

Predictor	Model 1 β (95% CI)	Model 1 *p*	Model 2 β (95% CI)	Model 2 *p*
Age (years)	−0.002 (−0.022 to 0.019)	0.88	−0.004 (−0.024 to 0.017)	0.712
BMI (kg/m^2^)	−0.013 (−0.088 to 0.062)	0.742	−0.005 (−0.072 to 0.063)	0.895
Diabetes mellitus	−0.461 (−1.049 to 0.127)	0.124	−0.385 (−0.965 to 0.195)	0.193
Duration since onset (months)	0.000 (−0.009 to 0.010)	0.972	0.001 (−0.008 to 0.010)	0.803
MAS (wrist flexors)	0.355 (0.175 to 0.535)	<0.001	0.238 (0.046 to 0.430)	0.015
Male sex	−0.202 (−0.656 to 0.252)	0.384	−0.113 (−0.531 to 0.306)	0.598
ΔWFR (hemi–contra)			1.371 (0.531 to 2.211)	0.001

Abbreviations: BMI, body mass index; CI, confidence interval; DSL, distal sensory latency; MAS, Modified Ashworth Scale; WFR, wrist-to-forearm ratio. Δ indicates hemiplegic side minus contralateral side. Robust (HC3) standard errors were used for inference.

**Table 5:** Model fit (adjusted R^2^) for base and extended regression models (with and without ΔWFR) predicting ΔDML and ΔDSL.

Outcome	Model	*N*	Adjusted R^2^
ΔDML	Model 1	85	0.251
ΔDML	Model 2 (+ΔWFR)	85	0.370
ΔDSL	Model 1	85	0.142
ΔDSL	Model 2 (+ΔWFR)	85	0.253

Abbreviations: DML, distal motor latency; DSL, distal sensory latency; WFR, wrist-to-forearm ratio. Adjusted R^2^ values are reported for each model.

## Data Availability

The data presented in this study are available on request from the corresponding author due to privacy and ethical restrictions related to patient data.

## Supplementary Materials

The following supporting information can be downloaded at: https://www.mdpi.com/article/10.3390/diagnostics16071088/s1, Table S1: Sensitivity analyses using ordered-category MAS and complementary linear mixed-effects models for bilateral median nerve latency outcomes; Table S2: Collinearity diagnostics and exploratory interaction analyses involving MAS and ΔWFR.

## Abbreviations

The following abbreviations are used in this manuscript:

MAS	Modified Ashworth Scale
NCS	Nerve Conduction Studies
DML	Distal Motor Latency
DSL	Distal Sensory Latency
CSA	Cross-Sectional Area
WFR	Wrist-to-Forearm Ratio
BMI	Body Mass Index
NIHSS	National Institutes of Health Stroke Scale
K-MBI	Korean version of the Modified Barthel Index
ΔDML	Hemiplegic minus contralateral Distal Motor Latency
ΔDSL	Hemiplegic minus contralateral Distal Sensory Latency
ΔWFR	Hemiplegic minus contralateral Wrist-to-Forearm Ratio
ΔCSA	Hemiplegic minus contralateral Cross-Sectional Area
IRB	Institutional Review Board
CI	Confidence Interval
