# Supplementary material for: Wrist Flexor Spasticity and Hemiplegic–Contralateral Median Nerve Latency Asymmetry After Stroke: A Bilateral Nerve Conduction and Ultrasound Study
Source: Diagnostics (Basel). 2026 Apr 3;16(7):1088. doi: 10.3390/diagnostics16071088 (PMC13073195; doi:10.3390/diagnostics16071088)
Supplement: Supplementary file 1 [file diagnostics-16-01088-s001.zip › diagnostics-4222628-supplementary.pdf]

Supplementary Tables

Supplementary Table S1. Sensitivity analyses using ordered-category MAS and complementary linear mixed-effects models for bilateral median nerve latency outcomes.

Outcome	Model	Term	β	95% CI	p-value
**Panel A. Ordered-category MAS sensitivity analyses**					
ΔDML	Base model	Ordered MAS	0.317	0.209 to 0.425	<0.001
ΔDML	Expanded model	Ordered MAS	0.230	0.110 to 0.349	<0.001
ΔDML	Expanded model	ΔWFR	1.430	0.764 to 2.096	<0.001
ΔDSL	Base model	Ordered MAS	0.252	0.116 to 0.388	<0.001
ΔDSL	Expanded model	Ordered MAS	0.166	0.021 to 0.312	0.025
ΔDSL	Expanded model	ΔWFR	1.396	0.567 to 2.225	0.001
**Panel B. Complementary linear mixed-effects models using raw bilateral latency values**					
DML	Mixed-effects model	Hemiplegic side	0.142	-0.110 to 0.394	0.271
DML	Mixed-effects model	MAS	-0.086	-0.190 to 0.018	0.105
DML	Mixed-effects model	Hemiplegic side × MAS	0.425	0.275 to 0.575	<0.001
DSL	Mixed-effects model	Hemiplegic side	0.376	0.089 to 0.663	0.010
DSL	Mixed-effects model	MAS	-0.075	-0.194 to 0.043	0.213
DSL	Mixed-effects model	Hemiplegic side × MAS	0.366	0.195 to 0.537	<0.001

Panel A shows HC3-robust multivariable linear regression sensitivity analyses in which MAS was re-specified as an ordered category corresponding to MAS 0, 1, 1+, 2, 3, and 4. Base models included MAS and covariates (age, sex, BMI, diabetes mellitus, and duration since onset), and expanded models additionally included ΔWFR. Panel B shows complementary linear mixed-effects models using raw bilateral latency values, with side (hemiplegic vs. contralateral), MAS, and a side-by-MAS interaction as fixed effects, a subject-level random intercept, and the same covariates as in the Δ-based models. β denotes the regression coefficient. Δ indicates hemiplegic minus contralateral.

Supplementary Table S2. Collinearity diagnostics and exploratory interaction analyses involving MAS and ΔWFR.

Panel	Variable / Term	Estimate	95% CI	p-value
**Panel A. Collinearity diagnostics**				
Correlation	Pearson r between MAS and ΔWFR	0.371		<0.001
Correlation	Spearman ρ between MAS and ΔWFR	0.333		0.002
VIF	MAS	1.193		
VIF	ΔWFR	1.203		
**Panel B. Exploratory interaction analyses**				
ΔDML model	MAS × ΔWFR	-0.326	-0.848 to 0.197	0.222
ΔDSL model	MAS × ΔWFR	-0.605	-1.198 to -0.012	0.046

Pearson and Spearman coefficients describe the association between MAS and ΔWFR. Variance inflation factors (VIFs) were calculated from the expanded Δ-based regression models and indicate low collinearity. Exploratory interaction analyses were performed by adding an MAS × ΔWFR interaction term to the expanded HC3-robust multivariable linear regression models for ΔDML and ΔDSL. Δ indicates hemiplegic minus contralateral.
